# Priority setting for maternal, newborn and child health in Uganda: a qualitative study evaluating actual practice

**DOI:** 10.1186/s12913-019-4170-6

**Published:** 2019-07-08

**Authors:** Lauren J. Wallace, Lydia Kapiriri

**Affiliations:** Department of Health, Aging and Society, McMaster University, KTH-236, Main Street West 1280, Hamilton, ON Canada

**Keywords:** Maternal, Newborn and child health, Priority setting evaluation, Low income countries

## Abstract

**Background:**

Despite continued investment, Maternal, Newborn and Child Health (MNCH) indicators in low and middle income countries have remained relatively poor. This could, in part, be explained by inadequate resources to adequately address these problems, inappropriate allocation of the available resources, or lack of implementation of the most effective interventions. Systematic priority setting and resource allocation could contribute to alleviating these limitations. There is a paucity of literature that follows through MNCH prioritization processes to implementation, making it difficult for policy makers to understand the impact of their decision-making on population health. The overall objective of this paper was to describe and evaluate priority setting for maternal, newborn and child health interventions in Uganda.

**Methods:**

Fifty-four key informant interviews and a review of policies and media reports were used to describe priority setting for MNCH in Uganda. Kapiriri and Martin’s conceptual framework was used to evaluate priority setting for MNCH.

**Results:**

There were three main prioritization exercises for maternal, newborn and child health in Uganda. The processes were participatory and were guided by explicit tools, evidence, and criteria, however, the public and the districts were insufficiently involved in the priority setting process. While there were conducive contextual factors including strong political support, implementation was constrained by the presence of competing actors, with varying priorities, an unequal allocation of resources between child health and maternal health interventions, limited financial and human resources, a weak health system and limited institutional capacity.

**Conclusions:**

Stronger institutional capacity at the Ministry of Health and equitable engagement of key stakeholders in decision-making processes, especially the public, and implementers, would improve understanding, satisfaction and compliance with the priority setting process. Availability of financial and human resources that are appropriately allocated would facilitate the implementation of well-developed policies.

**Electronic supplementary material:**

The online version of this article (10.1186/s12913-019-4170-6) contains supplementary material, which is available to authorized users.

## Background

In spite of significant renewed concern over women’s and children’s health over the last four decades, Maternal, Newborn and Child Health (MNCH) indicators have remained relatively poor, especially in Low and Middle Income Countries (LMICs) [1, 2]. According to the most recent burden of disease estimates, 5.8 million children under five, 2.6 million neonates and 275,300 women continue to die annually [2].

MNCH programs face numerous challenges, including competition between maternal and child health programs and actors, demands across the full continuum of care in contexts with scarce resources, and a lack of harmonization between MNCH and health systems development agendas. Furthermore, maternal and child deaths often take place in contexts of social and economic discrimination and marginalization; these contexts require inter-sectoral policy-making approaches that pay attention to upstream causes of mortality and morbidity in addition to clinically effective interventions [3, 4].

These challenges, in addition to the limited resources in LMICs, necessitate successful priority setting (PS) in order to ensure that the available resources are optimally used. In this paper we define priority setting interchangeably with resource allocation, and define it as the ordering and allocation of resources between competing programs or populations [5].

There is a limited body of literature on priority setting for MNCH programs at the global level [6–8] and at various levels of decision making in LMICs including the national [9–17] and sub-national levels [18–21]. At the global level, studies have focused on examining how priorities emerge. For instance, Smith & Shiffman use replicative process-tracing case studies to examine why neonatal and maternal health receive different levels of political attention and resources, despite the fact that they are both high-burden issues, while Deleye & Lang describe bilateral and private donors’ prioritization between different MNCH programs and their approaches to stakeholder engagement [6, 7]. Studies at the national and sub-national levels have mainly described priority setting criteria and approaches, stakeholders’ roles, and how the socio-political environment, such as political will, institutional capacity, and power dynamics between global, national and local actors may impact the prioritization process [9–17, 18–21].

For example Mayhew & Adjei, using a “policy priority” approach to examine national priority setting in Ghana for MNCH, found that traditional priority setting tools tend to consider benefits from disease specific interventions and are inadequate in capturing the benefits of important preventative interventions such as family planning [14]. In their examination of stakeholders’ participation in district level priority setting for Prevention of mother to child transmission (PMTCT) in Tanzania, Shayo and colleagues report that the ability of district health management teams to set locally-relevant priorities was limited due to the influence of donors in addition to the priorities at the national level [20].

However, there is a paucity of literature that follows the prioritization process through to implementation, while considering the political, sociocultural and economic context. Since good prioritization processes may not necessarily result in the desired health impact, it is of critical importance that policy makers are able to evaluate both their prioritization process and the impact of the prioritization decisions within their unique context. A holistic evaluation of priority setting for MNCH is an important area of analysis since the outcomes of a MNCH program not only impact public health at the individual and community level, but broader issues of health and development, such as health systems and gender equity.

This paper provides an evaluation of priority setting for MNCH in Uganda at the national and sub-national levels between 2010 and 2015, using Kapiriri & Martin’s conceptual framework for evaluating priority setting [5].

## Methods

### The analytical framework

There have been several efforts to develop frameworks to evaluate priority setting [22–25]. However, efforts have been mainly centered within the context of high income countries. For instance, Gibson and colleagues, through a case study of hospital operational planning in Toronto, identified quality indicators for successful priority setting [22]. Sibbald and colleagues used mixed methods with healthcare decision-makers, patients and caregivers, mainly in Norway, Canada, and the United Kingdom to identify and pilot test similar parameters [23, 24]. More recently, Barasa and colleagues developed a conceptual framework for evaluating macro and meso-level priorities by conducting a systematic review of all current literature in both high income and low-income settings. The parameters identified in their conceptual framework were similar to those proposed by Gibson and Sibbald [25].

In response to the need to better understand how best to evaluate priority setting in low resource settings, Kapiriri and Martin developed an evaluation framework based on the priority setting literature and Delphi interviews with global experts with experience in health prioritization in low- and middle-income countries [5]. Following its development, the framework underwent validation by both global experts in addition to policymakers and practitioners involved in health planning at the national and sub-national levels in Uganda [26]. It has been used to evaluate case studies of priority setting in Uganda [27–29].

Kapiriri and Martin’s framework was selected for use in this case study for its comprehensiveness and its relevance to priority setting in low resource contexts. Unlike existing frameworks, Kapiriri and Martin’s framework is informed by both the evaluation literature, including earlier frameworks, and the practical constraints facing low income countries, since it has been validated by decision-makers working in these settings. It also caters for political, economic and sociocultural issues which cannot be overlooked when examining priority setting in LMICs. Since the framework was validated by and for use in LICs, the authors assumed it was most appropriate for use in the study context.

Kapiriri and Martin’s framework identifies 5 domains, namely: contextual factors, pre-requisites, priority setting process, implementation and outcome and impact. The domains are all deemed critical to successful priority setting.

#### Contextual factors

It is difficult to comprehensively understand why priority setting is successful without recognizing the priority setting context. The political environment determines the extent to which priority setting processes are participatory and fair, the economic context impacts the availability of financial and human resources necessary for facilitating health sector priority setting and implementation, and the social and cultural context influences public acceptability of the priorities and their feasibility.

#### Pre-requisites

These include legitimacy and institutional capacity, political will and availability of resources. While legitimacy and institutional capacity ensure that the priority setting institutions and their decisions are respected by key stakeholders, political will and support can facilitate resource mobilization and allocation to the prioritization process and to the priorities [5].

#### The priority setting process

A successful priority setting process is:

(i) Participatory and based on clear and explicit processes, tools or methods;
(ii) Based on explicit and agreed on criteria: basing priorities on relevant and explicit context-specific criteria would improve consistency in priority setting and reflect public values (since the public is most affected by the priority setting process);
(iii) Fair: based on explicit relevant criteria, with mechanisms for publicizing the rationales and the decisions, appealing and revising the decisions, and enforcement [5, 30]. A fair process contributes to the legitimacy of both the process and the decisions and may increase stakeholder acceptability of the decisions;
(iv) Evidence based: using evidence in priority setting can discourage the potential influence of “politics” and increase the credibility of the decisions [5];
(v) Efficient: the investment (time and money) in the priority setting process should not exceed the benefits (which may include improved quality of decisions and stakeholder satisfaction and acceptance of decisions) [5].

#### Implementation

In health care priority setting, it may be a waste of resources if the identified priorities are never implemented. Implementation will only be successful if resources are available and allocated according to the identified priorities. This would result in decreased resource wastage. Another aspect of successful priority setting is its impact on stakeholders. Stakeholder involvement in the priority setting process or their exposure to publicized information (discussed above) would increase their understanding of and their recognition of the need for priority setting. Their involvement may also increase their satisfaction and compliance with the priority setting process and the outcomes; this would result in a reduction in complaints about the decisions.

#### Outcome and impact

According to Kapiriri & Martin’s framework, when the priority setting institution is transparent, stakeholders gain confidence in the priority setting process and the institution. This confidence can facilitate stakeholders’ compliance, thereby improving priority setting [5]. Furthermore, successful priority setting should facilitate the strengthening of the priority setting institution. This may occur via capacity building and/or increased investment in the health system. Successful priority setting should also enable the health system to achieve its goals of improved population health in terms of alleviating disease burden, reduction in health inequalities, and fair financial contribution. It should lead to improved financial and political accountability. Ultimately, successful priority setting ought to impact policy and practice [5]**.**

Each domain has a number of parameters and each parameter is associated with objectively verifiable indicator(s) (OVI) which permit different people, using the same measuring process, to obtain the same results independently, and means of verification (MOV), which specify the sources of evidence that will inform evaluators if an expected measure has been achieved (Table 1). Kapiriri and Martin’s evaluation framework informed the methods used for data collection and analysis in this study.

**Table 1:** Parameters for evaluating priority setting with corresponding means of verification and indicators

Parameters of Successful Priority Setting	Objectively Verifiable Indicators (OVI)	Means of Verification (MOV)
Contextual Factors		
Conducive political, economic, social and cultural context	Relevant contextual factors that may impact priority setting	Follow up intermittent interviews with local stakeholders, systematic longitudinal observations, relevant reports, media
Pre-requisites		
Political will	Degree to which politicians support the set priorities	Follow up intermittent interviews with local stakeholders, systematic longitudinal observations, relevant reports, media
Resources	Budgetary and human resource allocation to the health sector	National budget documents
Legitimate and credible priority-setting institutions	Degree to which the priority setting institution can set priorities; public confidence in the institution	Stakeholder and public interviews
Incentives	Material and financial incentives	National budget documents
The Priority Setting Process		
Stakeholder participation	Number of stakeholders participating, number of opportunities each stakeholder expresses opinion	Observations/minutes at meetings, media reports, special reports
Use of clear priority setting process/tool/methods	Documented priority setting process and/or use of priority setting framework	Observation/minutes at meetings, media reports, special reports
Use of explicit relevant priority setting criteria	Documented/articulated criteria	Observations/minutes at meetings, media reports, special reports
Use of evidence	Number of times available data is resourced/number of studies commissioned/strategies to collect relevant data	Observations/minutes at meetings, media reports, special reports
Reflection of public values	Number and type of members from the general public represented, how they are selected, number of times they get to express their opinion, proportion of decisions reflecting public values, documented strategy to enlist public values, number of studies commissioned to elicit public values	Observations/minutes at meetings, study reports, meeting minutes and strategic plans
Publicity of priorities and criteria	Number of times decisions and rationales appear in public documents	Media reports
Functional mechanisms for appealing the decisions	Number of decisions appealed, number of decisions revised	Observations/minutes at meetings, media reports, special reports
Functional mechanisms for enforcement	Number of cases of failure to adhere to priority-setting process reported	Observations/minutes at meetings, media reports, special reports
Efficiency of the priority-setting process	Proportion of meeting time spent on priority setting, number of decisions made on time	Observations/minutes at meetings, annual budget documents, health system reports
Implementation		
Allocation of resources according to priorities	Degree of alignment of resource allocation and agreed upon priorities, times budget is re-allocated from less prioritized to high prioritized areas, stakeholder satisfaction with decisions	Annual budget reports, evaluation documents
Decreased resource wastage	Proportion of budget unused, drug stock-outs	Budget documents, evaluation reports
Increased stakeholder understanding, satisfaction and compliance with the priority setting process	Number of stakeholders attending meetings, number of complaints from stakeholders, % stakeholders that can articulate the concepts used in priority setting and appreciate the need for priority setting	Observations/minutes at meetings, special reports, SH satisfaction survey, media reports, stakeholder interviews, evaluation reports
Decreased dissensions	Number of complaints from stakeholders	Meeting minutes, media reports
Improved internal accountability/reduced corruption	Number of publicized resource allocation decisions	Evaluation reports, stakeholder interviews, media reports
Strengthening of the priority setting institution	Indicators of increased efficiency, use of data, quality of decisions, appropriate resource allocation, % stakeholders with the capacity to set priorities	Training reports, evaluation reports, budget documents
Outcome/Impact		
Increased investment in the health sector and strengthening of the health care system	Proportion increase in the health budget, proportion increase in the retention of health workers, % of the public reporting satisfaction with the health care system	National budget allocation documents, human resources survey reports, interviews with stakeholders, media reports
Impact on institutional goals and objectives	% of institutional objectives met that are attributed to the priority setting process	Evaluation reports, special studies
Impact on health policy and practice	Changes in health policy to reflect identified priorities	Policy documents
Achievement of health system goals	% reduction in DALYs, % reduction of the gap between the lower and upper quintiles, % of poor populations spending more than 50% of their income on health care, % users who report satisfaction with the healthcare system	Ministry of Health documents, Demographic and Health Surveys, commissioned studies
Improved financial and political accountability	Number of publicized financial resource allocation decisions, number of corruption instances reported, % of the public reporting satisfaction with the process	Reports, media reports, interviews with stakeholders
Increased investment in the health sector and strengthening of the health care system	Proportion increase in the health budget, proportion increase in the retention of health workers, % of the public reporting satisfaction with the health care system	National budget allocation documents, human resources survey reports, interviews with stakeholders, media reports

Source: (Kapiriri, [26, 27]). This Table was originally published in BMC Health Serv Res

### Data collection

This was a qualitative prospective study involving a review of documents and key informant interviews. Data collection strategies were aligned with the means of verification proposed in the framework, where possible.

### Document review

BK (a Uganda based researcher) conducted the initial document review and LW supplemented the initial review by accessing policy documents not initially reviewed, as well as more recent media reports. The reviewed documents included policy documents and media reports that covered the period of 2010–2015. Policy documents included the *Health Sector Strategic and Investment Plans* (HSSIPs) II (2005/6–2009/10) and III (2010/11–2014/15), the *Roadmap for Accelerating the Reduction of Maternal and Neonatal Mortality and Morbidity in Uganda* and *A Promise Renewed: The Reproductive, Maternal, Newborn and Child Health Sharpened Plan for Uganda* [31–34]. These documents provided information on the documented national priorities and priority setting processes. The media reports (from the two main daily newspapers) were reviewed to assess the kind of MNCH priority setting information that is publically available.

#### Interviews

LK and BK conducted the interviews between 2013 and 2015 at the national and sub-national (district) levels. District level participants provided insight into the enablers and barriers to implementation. This study was reviewed and approved by the McMaster Research Ethics Board and the Makerere University School of Public Health Research Ethics Board. All respondents provided written, signed consent.

### Study sample and sampling strategy

#### Study setting

The study was conducted at the national level and at the sub-national level.

#### Sampling

This included both purposive and snowball sampling. The sampling frame included all stakeholders working within the health sector at the national and district level. Initially, stakeholders who were knowledgeable of priority setting for MNCH at the national level and sub- national levels were purposefully recruited. Initial respondents were identified through the internet, they were contacted via e-mail, which was followed up with a phone call. National respondents included Development Assistance Partners (DAPs), Researchers, Ministry of Health (MoH) officers and members of Civil Society organizations. District respondents included members of the district council as well as the district health team. Initial respondents were requested to identify additional knowledgeable respondents who were also interviewed. The snowball recruitment ended when no new themes emerged from the additional respondents. A total of 60 national level and 27 sub-national level respondents were recruited to participate in a wider study on health system priority setting. Of those, 54 stakeholders (38 at the national level and 16 at the sub-national level) discussed themes related to priority setting for MNCH. Of the 38 national level interviews, 12 were follow up interviews to assess whether the delayed parameters were successful.

#### Data collection

Data were collected using a semi-structured interview guide. The interview guide, which was based on the evaluation framework, was pretested for clarity with a group of LIC graduate students. Their comments were used in editing the interview guide (See Additional file 1). The interview guide was used with flexibility to allow the interviewer to follow up on any emerging themes. The interviews were conducted face to face by the principal investigator (LK) and the research assistant who had prior quantitative and qualitative research expertise and was specifically trained to ensure consistent data collection. Interviews lasted an average of 45 min and were audio recorded with permission from the respondents.

#### Data analysis and quality control

Interviews were transcribed verbatim and data were analyzed using Nvivo 10. To ensure quality control in coding the data, three members of the research team independently read and coded two interviews, developing a list of codes. In establishing codes, considerations were given to themes that were present in the majority (over 50%) of the interviews. The research team discussed their code names, resolved any discrepancies and developed an agreed upon code list which was used to analyze the data. Related codes were grouped into categories, each under a major node heading, with the individual sub-themes transformed into free nodes under these headings. Next, Kapiriri and Martin’s framework (Summarized in Table 1), was used to assess the degree to which the described process met the parameters for successful priority setting. Themes and sub-themes were summarized with representative quotes and linked with relevant parameters in Kapiriri and Martin’s framework to assess the degree to which the priority setting process was successful. The initial report was presented and validated at a knowledge exchange workshop with Ugandan policy makers–a type of member check.

## Results

This section is organized according to the parameters of successful priority setting that could be evaluated (summarized in Table 2). It was not possible to evaluate two of the parameters namely “decreased resource wastage”, and “increased public awareness of priority setting”, since we were unable to access the documents reporting how resources are allocated and there were no reports on public awareness studies, respectively.

**Table 2:** Evaluating priority setting for MNCH in Uganda using the parameters of successful priority setting

Parameters of Successful Priority Setting	MNCH Case Study
Contextual factors	
Conducive political, economic, social and cultural context	Low priority of the health sector and low education levels of the public posed challenges for implementation of priorities. Alignment with global priorities, especially the MDGs facilitated the prioritization and implementation of MNCH priorities.
Prerequisites	
Political will	Strong political commitment, especially with reference to MDG 4 and 5
Resources	Adequate resources for priority setting; funding for MNCH priorities increased during the period under review
Legitimate and credible priority-setting institutions	MNCH technical working groups have capacity and legitimacy
Incentives	Poor working conditions de-incentivized health workers for implementation
Priority setting process	
Stakeholder participation	Extent to which districts and the public are involved in priority setting unclear. Legitimacy of the role of politicians questioned.
Use of clear priority setting process/tool/methods	Tanahashi model, Lives Saved Tool and UN OneHealth Costing tool, BOD/CEA
Use of explicit relevant priority setting criteria	Equity, global priorities and calls, burden of disease, cost effectiveness
Use of evidence	Analysis of indicators and trends from UDHS, and HSSIPs, BOD, CEA, commodity profiles and coverage, and equity
Reflection of public values	No clear articulation of if/how public values considered. Some prioritization processes involved representatives of the public.
Publicity of priorities and criteria	Some MNCH plans and priorities publicized; No clear dissemination of the government’s rationale for prioritization.
Functional mechanisms for appealing the decisions	No mechanisms reported
Functional mechanisms for enforcement	No mechanisms reported
Efficiency of the priority-setting process	Inefficiencies in time spent developing multiple policies with similar priorities/messages with a lack of follow-up; delays in disbursements of funding to districts and in delivering reproductive health commodities to facilities
Implementation	
Allocation of resources according to priorities	Due to DAP influence, interventions aimed at child health reportedly received more resources than interventions related to maternal health, Implementation of child health related interventions and targeted reduction in child mortality were on track. Targeted drop in maternal mortality was well below the MDG target.
Decreased resource wastage	Not assessed
Increased stakeholder understanding, satisfaction and compliance with the priority setting process	Public and district representatives reported less understanding and satisfaction with the process. General dissatisfaction on part of all stakeholders with the outcomes of the process
Decreased dissentions	None reported
Outcome/Impact	
Improved internal accountability/reduced corruption	None reported.
Strengthening of the priority setting institution	See below
Increased investment in the health sector and strengthening of the health care system	Investment specific to MNCH increased over the period. MOH staff turnover a challenge
Impact on institutional goals and objectives	See below
Impact on health policy and practice	Three new policies formed to address MNCH, practice resulted in shifting service delivery to the hardest to reach and most burdened areas of the country
Achievement of health system goals	MDG 4 nearly achieved, MDG 5 not achieved; Fairness in financial contribution not reported; Response to public’s expectations could not be assessed
Improved financial and political accountability	None reported.

### Contextual factors

While the impact of the national political context was not discussed, respondents reflected on how a lack of prioritization of the health sector at the national level contributed to a lack of resources in the sector. Low education levels of the public also were described to pose challenges for priority setting. The discussion of the impact of these issues on priority setting is interwoven below.

#### Prerequisites to priority setting

The framework identifies four pre-requisites to a successful priority setting process: legitimate and credible priority setting institutions, political will, availability of resources and incentives.

##### Legitimate and credible priority setting institution

We found that the Ministry of Health is responsible for setting priorities. Priority setting for all health sector programs occurs every 5 years as part of the National health sector strategic and investment plan. Respondents felt that the MNCH technical working groups had the capacity and legitimacy to set MNCH priorities, possibly based on their qualifications and appointment.

##### Political will

Both the reviewed documents and interviews reported that there was a strong political commitment to MNCH, especially in relationship to achieving Millennium Development Goals (MDGs) 4 and 5 [33, 34]. Furthermore, the President Museveni is reported to have catalyzed the development of the Roadmap [33].

##### Availability of resources and incentives:

Both the document review and respondents’ discussions suggest that adequate resources existed for setting priorities but that there were inadequate funds for implementation, due to a small MoH budget. For instance, media reports also described how the government “slashed the health sector budget”, failing again to allocate 15% of monies to the health sector, in accordance with the Abuja declaration [35]. Although in general, media reports and respondents argued that the government’s budget for health is insufficient, a few respondents described how the funding specifically for MNCH increased during the period under review. Respondents discussed the government’s investment in the health system directed at health centres IV,^1^ as well as an increased allocation of funds for family planning:

*…so if you look at the funding we had for maternal health in the last five years, it has increased, especially with the commitment of the President on FP 2020, during the Family Planning Conference in London…(U_MoH_FU).*

*…there was recent investment in increasing the number of Health Centre IVs which would be in…geared towards increasing access to emergency and basic maternal obstetric and newborn care (U_DAP_FU)*

One respondent also discussed the government’s increased investment to recruit health workers into rural areas. Media reports suggested that in 2012 the government’s allocation for family planning increased from 3.3 billion Ugandan shillings per year to 5 billion Ugandan shillings a year for 5 years and that there was allocation of an additional 6.5 billion Ugandan shillings for recruitment of health workers in health centres III, IV [36, 37].

Respondents and media reports captured issues that contributed to de-incentivize key stakeholders, especially the implementers. For instance, a media report identified poor working conditions as one of the factors that have led to apathy about maternal mortality among health workers and managers [38]. One respondent reflects on the negative impact of a lack of incentives:

*“ …as long as we have as system whereby you are rewarded the same whether you perform or not, I think implementation is always going to remain a challenge…” (N_FU_18)*

#### The priority setting process

The key elements of a successful priority setting process are that it should be: (i) Participatory and based on clear and explicit processes, tools or methods, (ii) Evidence based and reflect public values, (iii) Fair: based on explicit relevant criteria, and have mechanisms for publicizing the rationales and the decisions, appealing and revising the decisions (based on new evidence), as well as public or legal leadership to ensure that the above fairness conditions are met [5, 30], (iv) efficient.

##### Stakeholder participation

Policy documents and key informants make reference to the involvement of specific stakeholders in the development of MNCH plans, these include Development Assistance Partners (DAPs), Politicians, Ministry of Health Officers, District Officers, Members of the Health Management Team (HMT) and health workers, and the public and their representatives.

The documents reviewed indicate that DAPs were involved in the development of plans by providing technical assistance [32, 34] or financial resources to the MoH [33]. Respondents also indicated that DAPs were involved in MNCH priority setting and participated in the annual sector review meetings that monitor implementation.

The most commonly cited politicians were Members of Parliament, who were reported to have been involved in the development of the Roadmap [33] and provided assistance in facilitating the implementation of both the Roadmap and the Sharpened Plan, and advocating for increasing the budget for health [33, 34]. These findings were corroborated by the newspaper review, which reported the President and the First Lady’s involvement in advocating for MNCH at local and international fora. For instance, the president was a keynote panelist at the 2012 London Summit on Family Planning while the first Lady was reported to have emphasized the important role of leaders and health workers in reducing Maternal and child mortality [39, 40].

Some respondents at both the district and national levels were sceptical of the contribution of politicians. Respondents at the national level explained how politicians (and politics) could negatively impact both priority setting and implementation. Within districts, politicians were reported to have the tendency to respond to public pressure by, for example, constructing health units in specific locations to secure votes. At the national level, due to personal interests, politicians were described to make public pledges or favour specific interventions that may not necessarily be backed by evidence:

*“...Listen I just recently, when the President gave his State of the Nation address I asked the assistant of the Minister of Health... He said the President is going to say things there that are going to become policy. So I said oh so that is the person making it for you. That’s one way they do it…”* (N_MNCH_18).

Stakeholders from the MoH and the district were identified as the most legitimate in setting health sector priorities. The document review and interviews with respondents suggest that these stakeholders were involved in the development of all three of the key MNCH policies and the HSSIPs II and III [32–34]. A respondent commenting on district stakeholders’ participation in national priority setting processes said:

*“…Right from the national level the Ministry of Health engages with different stakeholders especially at the review meetings…which also bring on board district level to highlight the exact regional challenges people are having and to highlight the specifics that need to be addressed at district level…” (N_MNCH_16)*

Another group of district level stakeholders, identified in the MNCH policies and interviews, were members of the HMT and health workers. Both were involved in the development of the HSSIP III, while professional bodies participated in the development of the Sharpened Plan [32, 34]. These findings were corroborated by some of the respondents. However, the reviewed documents did not discuss the involvement of the HMT, health workers or their professional bodies in the development of the Roadmap [33].

While district-level respondents recognized that national-level priorities such as maternal health also reflected local issues, they expressed the need for more discretion and flexibility to tailor the funds received from the MoH to reflect the situation on the ground:

*“…I feel since we are local government we need to be given a lot more discretion when it comes to priority setting. So that we know what is peculiar in terms of [district], where we need to put more money or more emphasis…at sector level we can say maternal health is no longer a big problem let us put more money maybe in sorting out the issue of jiggers or something of the kind. So we feel we should be given discretion…”* (D_M5).

The last group of stakeholders was the public. The public, or their representatives, are reported to have been involved in the development of the reviewed documents [32, 33]. This was echoed by some of the national level respondents. However, some respondents at both national and district levels argued that citizens need to be empowered more to participate in the selection of priorities. For instance, one respondent stated:

*“...our communities are not empowered to be able to contribute to priority setting…Right now communities are waiting as if government is doing them a favour, they don’t demand, a patient dies in a hospital, they go and thank the doctors you know when they should be actually pushing…Who is going to lead that revolution? Even NGOs don’t go that far…”(N_MNCH_18).*

Insufficient involvement of the public in priority setting processes was thought to contribute to citizens’ apathy and lack of support for successful implementation of MNCH programs:

*“…Right from the word go there are people whom we don’t involve. Who may be key players during implementation …For example we have seen that in [our] district women who come to deliver in health facilities are very few…But at the end of the day they are people we are leaving out the very people we expect to come to the facilities. They don’t turn up at the end of the day. So I think that could be one of the reasons. Yea. So (the reason) why we are not successful implementing is basically involvement…”(D_T8)*

##### Use of clear priority setting tools/methods

National level priority setting is reportedly guided by the Burden of Disease (BOD)/Cost Effectiveness Analysis (CEA) approach. Subsequent MNCH processes used tools such as bottleneck analysis derived from the Tanahashi model, the Lives saved tool (LiST) and the UN OneHealth costing tool [34].

##### Use of explicit relevant priority setting criteria

We identified several considerations, namely: alignment with global priorities, equity, and evidence. A cross cutting idea in all the reviewed policy documents and in media reports was that the MNCH priorities selected were in line with, or based on, global or regional agendas, particularly MDGs 4 and 5. For example, the Roadmap was developed in response to a special session of the conference of African Union Ministers of Health in Maputo in 2006 [33] while the Sharpened Plan was catalyzed by the Child Survival Call to Action in Washington in 2010 [34]. The influence of global priorities was also captured in respondents’ discussions, as expressed by a respondent:

*“…But at the end of the day it [MNCH] is a very visible thing in Uganda, at global level, at regional level, MNCH is huge, huge, huge priority. Lots of money has been pouring in from every direction for MNCH in the country…” (N_HS_FU)*

While some respondents viewed global influence as relevant for national priority setting, others felt that global pledges for action may cause unnecessary duplicity. For instance, one individual reported on the influence of the “promise renewed” pledge in 2012 on the development of an unnecessary “Sharpened” MNCH plan:

*“...So from there was no need for a plan to be honest, the road map that we had was already quite comprehensive and was not even implemented …So I don’t know whether you know how this sharpened plan came up, it is from a global movement…”(N_MNCH_8)*

Another criteria that was discussed was equity. All of the MNCH policies discuss the need to improve equitable access to healthcare and target the most vulnerable and disadvantaged groups, including women and children, especially those in remote areas and ethnic minorities [32–34]. Equity is also seen by respondents as central to the prioritization of MNCH, as mothers and children and rural dwellings, are understood to be vulnerable. As explained by a couple of national level respondents:

*“…Yes. So, when we talk about vulnerable population, most of the time we think about women, children, elderly… yeah, and adolescents…” (N*_*EM_6)*

*“… Equity, access, women, children, you know, the poor, rural/urban differentiation…all of these are things that for sure guide our priority setting…” (N_MNCH_19)*

District-level respondents described equity in terms of fairness in the distribution of resources; as explained by one respondent, fairness is considered in the distribution of maternity wards between the sub-counties within the district*:*

*“…So, if you are building a maternity ward in sub-county A and there is no maternity ward in sub-county X, you focus [on the sub-county without a ward]...That’s how we have managed to have maternity wards in all the sub-counties, because we want to promote fairness …and equity…” [D_T_20].*

##### Use of evidence

The documents revealed that various types of evidence were used, such as MNCH indicators and trends, including an equity analysis, burden of disease, and cost-effectiveness. To a lesser extent, policies stated that MNCH commodity profiles and analyses of factors that limit the attainment of adequate coverage and “global evidence and practice” also informed the priority setting process. This evidence was obtained from Uganda Demographic and Health Surveys (UDHS) and previous health and MNCH policies [33, 34].

These findings were supported by the respondents, who for example, discussed the way that evidence is used to identify the most vulnerable populations and regions:

*“…the UDHS showed us the indicators for example family planning and uptake of skilled care…. and it became clear that some regions were more at a disadvantage than others…” (N_MNCH_3).*

##### Reflection of public values

We found no clear articulation of how the public values were considered in the prioritization process. However, some of the prioritization processes directly involved representatives from the public (e.g. civil society and religious groups). It is also possible that public values were enlisted and presented with the findings from the situation analysis which was conducted prior to program implementation.

##### Publicity of priorities and criteria

All the policy documents and MNCH specific reports are available online. The degree to which these are accessible to the public is difficult to assess. Furthermore, MNCH-related issues were discussed in the media. Some of the issues that were highly publicized included the dissemination of policy documents, as in the case of the Roadmap, the government’s focus on reducing Maternal and Child Mortality to achieve MDGs 4 and 5, prioritization of family planning, antenatal care, human resources including skilled attendance at birth, PMTCT, and Insecticide Treated Nets (ITNs), and emergency obstetric care [36, 41, 42]. Most of these were publicized following major political announcements, however, newspapers did not consistently publicize the government’s rationale for prioritization. Where a rationale was provided, choice of interventions or diseases was often linked to the goal of reducing mortality to achieve MDG 4 and 5. Respondents did not discuss publicity in relationship to MNCH specifically.

##### Functional mechanisms for appealing the decisions and for enforcement

This study did not identify any formal explicit appeals and revisions mechanisms or mechanisms for enforcement. Complaints were channeled through the media, for example, grievances about health systems challenges such as stock-outs of reproductive health commodities were reported [43, 44].

##### Efficiency of the priority setting process

There was a consensus among respondents that there were major challenges with following up on plans to implement the priorities. Some respondents argued that this renders the time spent developing plans wasted, as explained by one respondent in the case of the Roadmap:

*“… We had a road map and we still have a road map for accelerated reduction of maternal/newborn mortality which is non-sectoral highlighting everybody’s roles and responsibilities. And we had a wide consultation you know bringing together the health group then calling on the other ministries. There had been meetings organized and [paid for by] the Ministry of Finance…There has not been adequate follow up on this process. And we believe that unless it is picked up again, I believe it will be difficult for us to address maternal health…”(N_MNCH_16).*

Furthermore, some respondents discussed the detrimental impact of delays in the MoH’s release of funds to districts for implementation. Media reports also discussed inefficiencies created by supply chain bottlenecks, which delayed the delivery of commodities such as mama kits to health facilities or neglected to account for kits in storage, wasting funds [44, 45]. The production of several documents identifying MNCH priorities was also an inefficiency highlighted by respondents.

#### Implementation

The following parameters may indicate successful implementation; (i) Allocation of resources according to priorities, (ii) Decreased resource wastage, (iii) Increased stakeholder understanding, satisfaction and compliance with the priority setting process, (iv) Decreased dissensions, (v) Improved internal accountability/reduced corruption and (vi) Strengthening of the priority setting institution.

##### Allocation of resources according to priorities

Among some respondents there was a sense that resource allocation between MNCH interventions had been uneven, with some programs such as HIV and child health (malaria and immunization), being allocated more resources than maternal interventions. This was, in part, attributed to external funding, as explained by a participant working with civil society who described how external funding led to a distortion of the original HSSIP, resulting in more resources being availed for child health programs compared to reproductive health issues:

*“…when it comes to implementation (that) is when you see Global Fund coming in with their money, those who are focusing on malaria, child (health), TB, (then) there is a distortion of the original HSSP…”(U_MNCH_1)*

##### Stakeholder understanding, satisfaction and compliance with the priority setting process

As discussed above, several stakeholders are involved in the national level priority setting process. Those who are directly involved (such as the MoH officers) were likely to report understanding and satisfaction with the process compared to those who were not involved (for example, district level respondents, and the public). With regard to the public, limited involvement and lack of publicity mechanisms makes it difficult for them to access the prioritization processes and related information. Low literacy levels were thought to limit the public’s understanding. Respondents thought that this may explain the public’s questioning or resistance to interventions such as facility-based births, family planning, immunization, and the non-use of ITNs, for instance:

*“…on the other side even the users are not adequately educated. For instance the mosquito nets …Some use them for other things…some keep them in their houses and don’t use them at all…”(N_NCD_17)*

Some respondents indicated that while some stakeholders seemed fairly satisfied with the priority setting process, there was general dissatisfaction with the outcomes of the process, especially since declines in mortality were less pronounced for neonates and mothers than for children. Respondents also decried the weak health infrastructure, poor motivation of staff and lack of essential commodities and their dissatisfaction with the negative impact of donor funding on implementation. In the media, the public also reported their dissatisfaction with the poor implementation of priorities, including the lack of achievement of MDG 5, and the impact of the weak health system, as well as the negligence of medical staff and the impact of their behaviour on maternal deaths [43, 46].

##### Decreased dissensions

While there might be increased dissensions in the initial stages of systematic and transparent priority setting processes, dissensions should decrease when other critical parameters such as publicity and stakeholder involvement are addressed. No specific complaints about the priorities were aired.

### Outcome/impact

Successful priority setting could have the following outcomes/impacts (i) Increased investment in the health sector and strengthening of the health care system, (ii) Impact on institutional goals and objectives, (iii) Impact on health policy and practice, (iv) Achievement of health system goals, (v) Improved financial and political accountability and (vi) Increased investment in the health sector and strengthening of the health care system.

While some contexts have designated priority setting institutions, Uganda’s Ministry of Health is charged with setting health priorities. Hence, while the framework separates parameters related to the Ministry of Health from those related to the priority setting institution, we combine them in the case of Uganda.

#### Improved internal accountability/reduced corruption

No instances of corruption or issues with internal accountability in relationship to MNCH were reported.

#### Increased investment in the health sector and strengthening of the healthcare system

We found that funding for MNCH increased during the period under review. However, it was not possible to assess the degree to which this, or the priority setting process contributed to health system strengthening since both the media and interview findings indicated a weak health system. Two respondents attributed this to the high rate of turnover in key positions such as officers in the Ministry of Health during the period under review:

*“…if I take governance…I think over the last few years we’ve had a bit of challenges in the Ministry of Health where many Ministers have changed…there has been a change of Ministers…I must say, quite frequently. But also many critical people, like experienced planners, left the Ministry for one reason or another…”(N_MNCH_FU8).*

Some of the participants thought that better institutional capacity in the MoH would improve the stewardship of resources, improve the allocation of resources, and reduce donors’ negative influence. For instance, one respondent partially attributed the negative influence of DAPs on priority setting to the weakened institutional capacity of the MoH, which rendered it unable to balance the interests of various stakeholders:

*“... Please understand me very well I don’t blame donors or NGOs. No. The problem is leadership…the lack of it…. And then they find a system with inadequate leadership that’s when they [DAPs] try to do their thing...”(U_MCH_18).*

### Achievement of health system goals

Both the reviewed documents and the interviews indicated that Uganda achieved the targeted reduction in neonatal mortality, and nearly achieved the targeted reduction in child mortality. As evidenced by the most recent Demographic and Health Survey, while the implementation of child health related interventions were on track, including coverage of ITNs, Diphtheria, Pertussis and Tetanus (DPT) III, measles vaccination, and pregnant women accessing comprehensive PMTCT, progress in reducing the maternal mortality ratio was slow [47]. Although the overall drop in maternal mortality was well below the MDG target, the targeted contraceptive prevalence rate was achieved, and considerable gains were made with regard to other important reproductive health targets including health facility deliveries, skilled attendance, and four antenatal care visits.

Several respondents attributed the successful implementation of key child health interventions to the support of development assistance partners:

*“…the child health component has been…it will be well implemented…quite an improvement which is related to external funding. Yeah because especially…GAVI came in with support for immunization…which we didn’t have before…Then Global Fund came in with their anti-malaria drugs…[and] the ITNs…Of course the government contributed and has been contributing to like purchase of vaccines…but getting this global support made a difference to improve our coverage and quality…”(N_MNCH_8).*

Uganda’s most recent DHS [47] indicates that while Uganda nearly achieved the under 5 mortality rate, and achieved the neonatal mortality target, progress to reduce the maternal mortality ratio was slow. It was not possible to assess the extent to which fairness in financial contribution and the response to the public’s expectations were improved.

#### Impact on health policy and practice

During the period under review (2010–2015), three new policies [32–34] was formed as a result of priority setting for MNCH. As a result of the policy changes, especially the sharpened plan, service delivery was shifted to focus geographically on the hardest to reach and most burdened areas of the country. However, it may be too early to assess the impact of this shift on practice and on Maternal Mortality.

#### Improved financial and political accountability

No issues with financial accountability were reported by respondents. With regard to political accountability, as discussed above, the extent to which public values were actually reflected in the priority setting process is difficult to ascertain.

## Discussion

This paper describes and evaluates priority setting for MNCH in Uganda using Kapiriri and Martin’s framework [5]. To our knowledge, this is one of the first case studies that follows through the MNCH prioritization process to implementation at the national level. The framework enabled us to assess the degree to which priority setting for MNCH has been successful, although there were parameters which could not be assessed since their means of verification were beyond the strategies used in the current study (see Table 2). While this study draws on a Ugandan case study, the social features of the Ugandan context, including the national and sub-national decision-making dynamics are applicable in many other LMIC settings.

Consistent with other studies, the social, cultural, economic and political context impacted priority setting [6, 10, 13, 17, 18, 20, 48]. Issues such as alignment with global priorities, specifically MDG 4 and 5, strong political will and donor interests were some of the key factors that contributed to resource mobilization and the prioritization and implementation of MNCH priorities, especially the child health related interventions.

The evaluation of the parameters related to the prioritization process revealed mixed findings, whereby the process was deemed successful in some aspects and not in others. The output of a successful prioritization process is a list of *feasible* and appropriate priorities. We found that there were at least three prioritization processes that occurred during or overlapped with the period of 2010–2015 that resulted in three main documents and lists of priorities [32–34]. While consistent in the overarching goal of attaining MDGs 4 and 5, there were subtle differences in the priorities, strategies, targets and indicators adopted in each plan. The existence of multiple lists of MNCH priorities may be problematic since it reflects duplicity of efforts and resources that could have been better allocated elsewhere in the health system. Although some have argued that MNCH programs face unique priority setting challenges that require safeguarding within a health systems approach [14] the lack of harmonization between MNCH programs and actors and health systems development agendas has frequently been discussed in the literature [3]. Ideally, there should be one prioritization process, and list which all stakeholders buy into and monitor and evaluate.

Notably, and consistent with the literature evaluating the fairness of a priority setting processes, the process fell short [49]. The findings that there was limited participation by the public and district-level stakeholders and lack of explicit appeals and enforcement mechanisms are consistent with findings from studies conducted in similar contexts [20, 50]. The lack of sufficient participation of the public, in particular, has been attributed to low literacy levels, for instance [49]. Some have blamed the limited involvement of the districts on decentralization, which, contrary to its intention, is thought in some contexts, including Uganda to promote top-down power structures, rather than facilitate bottom-up planning [20, 50]. Croke and colleagues argue that in Uganda, the politically-motivated creation of new districts has resulted in district health management teams without adequate capacity and resources [50].

It was beyond the scope of this project to assess whether the identified priorities were feasible and if the allocation of resources were aligned with the identified priorities, however, the literature indicates that more attention and resources seemed to be focused towards the implementation of child health related priorities than other priorities such as the reduction of maternal mortality. Studies suggest that child health interventions such as vaccines are fairly simple, inexpensive, evidence-based solutions that can be addressed effectively and cheaply and therefore have generated considerable donor interest [51]. Arguably, allocating resources according to the identified priorities would contribute to their successful implementation and subsequent positive health outcomes [5]. Although studies suggest that financial resources can positively impact health sector agenda setting in LMICs, numerous studies also show that financial resources have the power to draw attention to some priorities while neglecting others [6, 48]. This is demonstrated by our analysis, which suggests that resource allocation to child health may have contributed to the achievement of MDG 4 as compared to the lack of achievement of MDG 5. The shortfall in meeting the maternal mortality target was a source of stakeholder dissatisfaction and was also blamed, in part, on a weak health system. However, while interventions within the health sector contribute to maternal mortality shortfalls, they do not explain them entirely, since maternal mortality is determined by social and economic determinants of health such as education levels, socio-economic status, gender inequalities and cultural factors that also requires inter-sectoral action by actors outside of the health sector [3, 4].

The overall impact of priority setting for MNCH on the health system was difficult to assess. However, based on the findings that some of the programs within MNCH such as family planning received additional funding, it can be inferred that there was increased investment in, and hence, strengthening of the health sector. Studies suggest that strong institutions are more likely to carry out priority setting successfully [5]. However, although invested in the health sector, such funding that is attached to specific programs and interventions, such as donor funding, may have negative repercussions such as fragmentation of the system as well as reduced government funding [3, 52]. Although there were investments made in MNCH over the period, another issue which may have negatively impacted the health system was the weak institutional capacity of the Ministry of Health, due to high rates of staff turnover. In an earlier study, Croke attributes high rates of turnover in Uganda’s Ministry of Health to the involvement of MoH leaders in corruption scandals which have de-incentivized workers in the MoH and led highly qualified skilled personnel to leave their posts [50].

While it was possible to evaluate almost all the parameters within the framework, it was not possible to obtain information on some of the parameters. This could point to the sensitivity of some of the parameters e.g. resource wastage, which could be highly politicized. Such politicized issues may not be documented and may have been too sensitive to be discussed in an interview. The lack of information on some of the parameters could also be due to the proposed means of verification (MOV), e.g. real time meeting observations, public surveys which were beyond the scope of the study. This limitation points to the need for (i) using additional approaches such as focus group discussions where sensitive issues can be explored, (ii) integrating evaluation within the prioritization cycle to ensure that resources are available for some of the special data collection (e.g. public surveys) which may be necessary. Integration of evaluation would also facilitate the collection of the relevant information in real time without additional resources e.g. for real time meeting observations.

### Study limitations

The findings from this study should be interpreted with caution. First, the findings may not be generalizable, as is the case in all qualitative studies. Moreover, while sampling stakeholders with knowledge of the study phenomenon reflects their lived experiences with priority setting, it is not possible to rule out the potential for reporting bias. Third, while we endeavored to recruit all knowledgeable respondents, purposeful sampling, coupled with theoretical snowball sampling may have introduced bias, the sample may not be representative of all knowledgeable stakeholders. However, the triangulation of the sources of information (interviews and documents at the national and district levels) as well as recruiting a wide variety of respondents may serve to mitigate selection bias and improve the validity and generalizability of the results. Lastly, we were unable to evaluate all the parameters within the framework due to the methods employed in this study. These parameters required interviews with the public; this approach was beyond the scope of this paper. While this may affect our conclusions about the specific parameters, the degree of independence between the parameters still allows us to make credible conclusions.

Finally, rather than using a completely grounded approach to examine this case study, the authors used a specific framework for evaluating priority setting; the chosen conceptual framework informed the questions and prompts used in interviews and framed data analysis. There is a possibility that the choice of another conceptual framework may have produced slightly different findings. However, this is unlikely since the framework was developed through a rigorous method that comprehensively incorporates both issues noted in the literature and the experiences of practitioners, and is therefore holistic.

## Conclusion

The global focus on MNCH has led to its prioritization in Uganda. The formal process is participatory, and the program has garnered financial support, which may have contributed to strengthening of the health sector. Particularly, more investment in and focus on child health interventions may have contributed to positive child health outcomes. However, the multiple prioritization processes, lack of engagement of the public and district level stakeholders, limited transparency about the decisions and the criteria used, and lack of appeals and enforcement mechanisms impacted the fairness of the process. Furthermore, lack of means to follow through with some of the priorities limited their implementation.

To strengthen prioritization for MNCH, the legitimacy of the MoH should be strengthened by all actors in MNCH (including DAPs, the public and district officers) participating in the MoH prioritization process and adhering to the identified priorities. There should also be an internal or external body that will hold the stakeholders accountable and deal with any dissensions. All partners should commit to investing their resources according to the identified priorities. The nature of maternal health necessitates horizontal, inter-sectoral planning that takes into account the need for harmonization between MNCH, health systems development agendas, and the social determinants of health.

Monitoring and evaluation of both the priority setting process and the outcomes would contribute to identifying areas for improvement. Kapiriri and Martin’s framework and proposed sources of information provided could provide viable guidance for evaluating priority setting processes for MNCH. The framework is flexible enough to be used to investigate the specific facilitators and constraints to priority setting processes within a particular program such as MNCH, keeping program or topic specific needs visible, while measuring success based on the development and strengthening of a well-functioning health system.

## Additional file

Additional file 1: Interview guide. (DOCX 26 kb)

## Data Availability

The datasets generated and/or analysed during the current study are not publicly available because the nature of qualitative research makes it impossible for us to avail the data without compromising the participants’ confidentiality; but could be available from the corresponding author on reasonable request.

## Abbreviations

BOD: Burden of Disease
CEA: Cost Effectiveness Analysis
DAP: Development Assistance Partner
DHS: Demographic and Health Survey
DPT: Diphtheria, Pertussis and Tetanus
GBD: Global Burden of Disease
HSSIPs: Health Sector Strategic and Investment Plan
ITN: Insecticide Treated Net
LMIC: Low and Middle Income Country
MDG: Millennium Development Goal
MNCH: Maternal, Newborn and Child Health
MoH: Ministry of Health
PMNCH: Partnership for Maternal, Newborn and Child Health
PMTCT: Prevention of Mother to Child Transmission

## Footnotes

1 The Uganda health system is decentralized. Within the decentralized framework, there are five levels of care in a district: Level I corresponds to the village health team who are the first point contact of contact between the patients and the healthcare system. Level II corresponds to Health Centre II which provides outpatient, antenatal immunization and outreach service. Level III corresponds to Health center III which provides all services provided at HC II, PLUS inpatient care and environment health. Level IV corresponds to HC IV which provides all services provided at HC III PLUS surgery and blood transfusion and Level V corresponds to the General Hospital which provides all services provided at HC IV, ambulatory and in patient services, emergency obstetrical care and caesarean section, in addition to the basic care in the four main disciplines. These hospitals can also have a limited number of specialists and function as referral units for the Health Centre IV.
