# Supplementary material for: Priority setting for maternal, newborn and child health in Uganda: a qualitative study evaluating actual practice
Source: BMC Health Serv Res. 2019 Jul 8;19:465. doi: 10.1186/s12913-019-4170-6 (PMC6615092; doi:10.1186/s12913-019-4170-6)
Supplement: Supplementary file 1 — Interview guide. (DOCX 26 kb) [file 12913_2019_4170_MOESM1_ESM.docx]

**INTERVIEW GUIDE FOR THE RETROSPECTIVE INTERVIEWS WITH POLICY MAKERS (MNCH Theme officer)**

**Introductory questions**

1. Please introduce yourself and talk about what your roles are within the ministry of health? (probe for role in priority setting)
2. Priority setting is sometimes referred to as resource allocation—the process through resources are allocated between competing programs or diseases. For purposes of this research, we will focus on human and financial resources. Please tell me more about your experiences with this process within the ministry of health? Who is involved, how and why?
3. How successful would you say that the most recent priority setting process was? Please give reasons for your response.—what criteria did you use in coming to this conclusion
4. What factors contributed to the success/ failure of priority setting? Probe for contextual factors (Social, cultural, economic, institutional capacity, availability of resources and incentives)

**Top five priorities comparison**

*We will now talk about the top priorities for the ministry of health and how these compare to some selected conditions*

1. What, in your opinion, are the top five health problems at the national level? (How were these identified?)
2. I understand that after the HSSP 5 year national priorities, annually priorities are set within those set in the HSSP. Would you please describe this secondary annual process?
3. Choosing the top priority… and thinking about the range of decisions you have just described;
   1. Who has been involved in decision-making? Why was this person/ organization/ dept etc involved? How were they involved?
   2. Please describe how (the process) decisions about these priorities were made?
   3. What factors have influenced PS with respect to these top five priorities, in this institution? (Probe for criteria, used of evidence)What has been the nature of the influence? The outcomes?
4. In your opinion, how do the top five priorities compare to the level of priority put on Epidemics e.g. HINI/Ebola; HIV, Maternal, newborn and child health (MNCH), new vaccines and non- communicable diseases ? (Probe for reasons)
5. How would **YOU** rank the above conditions relative to the five top national priorities? Please explain your ranking.
6. What are your perceptions about the effects the above conditions may have (if any) on priority setting within the health system?

*We will now specifically talk about priority setting within your specific program MNCH;*

1. Considering MNCH programming; what are the priorities you are focusing on?
2. How were these priorities identified? Probe for:
   1. What process and/or framework was used to identify the priorities
   2. What criteria were considered?
   3. What stakeholders were involved in the prioritization process?
   4. What evidence was used?
   5. Were the decisions publicized?
   6. What happens if someone disagrees with the decisions that were made?
3. To what extent have these priorities been successfully implemented? (Please give reasons for the response)
4. What have been the impact of the prioritization process on: the stakeholders- including the public, the priority setting institution, and the ministry of health?
5. What are the main enablers/ constraints in implementing the priorities in your program? (Probe for Social, cultural, economic, institutional capacity, availability of resources and incentives)

**Wrap Up**

1. As a policy maker, what would say are the most urgent or difficult problem(s) with regards to priority setting at the national level?
2. Do you have any additional information or questions you would like to raise?
3. Would it be ok to contact you at a later date should there be issues that need to be clarified?

**Thank you for your time**
